# Optimization of brewing conditions for *Tieguanyin* oolong tea by quadratic orthogonal regression design

**DOI:** 10.1038/s41538-022-00141-7

**Published:** 2022-04-25

**Authors:** Qing-Qing Cao, Jie-Qiong Wang, Jian-Xin Chen, Fang Wang, Ying Gao, Daniel Granato, Xuebo Zhang, Jun-Feng Yin, Yong-Quan Xu

**Affiliations:** 1 Tea Research Institute Chinese Academy of Agricultural Sciences, Key Laboratory of Tea Biology and Resources Utilization, Ministry of Agriculture, 9 South Meiling Road, 310008 Hangzhou, China; 2 Graduate School of Chinese Academy of Agricultural Sciences, 100081 Beijing, China; 3 Department of Biological Sciences, Faculty of Science and Engineering, University of Limerick, V94 T9PX Limerick, Ireland; 4 National Tea Quality Supervision and Inspection Center (Fujian), Anxi, 362400 Quanzhou, China

**Keywords:** Agriculture, Nutrition

## Abstract

*Tieguanyin* is one of the most consumed oolong teas because of its distinctive flavor. The brewing process is crucial for the flavor performance of traditional teas, thus the effects of brewing conditions, including water/tea ratio (R), brewing temperature (T), and time (S) on the sensory traits, chemical composition, and antioxidant activity of *Tieguanyin* tea infusion were investigated using quadratic orthogonal regression design. Results showed that R affected all the quality variables most, its reduction could lead to the promotion of tea infusion concentration, antioxidant activity, and taste intensity, which was favored by the tea consumers drinking tea almost daily (DTD) but unacceptable for those drinking tea hardly (DTH). Based on the optimization of brewing conditions in response surface methodology (RSM), we recommended several brewing schemes for diverse consume goals: R = 34 mL/g, T = 80 °C, S = 80 s for DTH; R = 39 mL/g, T = 100 °C, S = 127 s for DTO (the consumers drinking tea occasionally); R = 20 mL/g, T = 100 °C, S = 100 s for DTD; R = 26 mL/g, T = 100 °C and S = 127 s for the common consumers seeking for flavor and health benefits. These results would be helpful for tea consumers with multiple demands.

## Introduction

Green, black, yellow, oolong, white, and dark tea, are recognized as the six types of traditional Chinese teas, which are classified by the processing technology and fermentation degree^1^. Oolong tea is a semi-fermented tea whereby. The *Tieguanyin* variety, originating from southern Fujian in China, is one of the most widely consumed oolong teas due to its distinctive flavor, including floral fragrance, refreshing, and mellow mouthfeel^2^.

Brewing is an essential process to release flavor compounds from tea leaves and its main factors include the selection of brewing water and method, water/tea ratio, brewing temperature, and time. Water plays a crucial role during tea brewing, and pure water with lower pH and mineral content has been reported to be the best choice in most situations^3,4^. Brewing condition affects the sensory profile, phytochemical properties, and antioxidant activity of different teas, such as green^5–7^, oolong^8,9^, and white teas^10,11^. Based on flavor and health benefits, the optimizations of brewing conditions for green^12^ and black teas^13^ have been studied thoroughly to support industrial activities and to provide scientific guidance for consumers. Nonetheless, it’s worth noting that, in both studies, powder leaves were used for the extraction procedure (i.e., brewing) and the sensory analyses were performed by a trained panel, which are reasonable for research purposes but somewhat different from what for the rituals adopted by ordinary tea consumers. Certainly, it’s extremely important to understand the consumer expectations and emotions for food designers or manufacturers. However, related studies from the standpoint of consumers are extremely rare. What’s more, there was no study involving the optimization of the brewing condition of *Tieguanyin* oolong tea.

Quadratic orthogonal regression design is an experimental design method of a multivariate statistical analysis based on response surface methodology (RSM). It has been fully verified to have a high superiority in the research of quantitative relationships among multiple variables, and applied widely in agrotechny, engineering, biology, and many other fields, to obtain an optimum technology parameter^14–16^.

In this study, a quadratic orthogonal regression design was used to investigate the effects of brewing factors, including water/tea ratio (R), brewing temperature (T), and time (S), on the sensory traits, chemical composition, and antioxidant activity of *Tieguanyin* oolong tea infusion. We divided consumers into three groups, that’s DTH (the consumers drinking tea hardly, i.e., 0–1 days/week), DTO (the consumers drinking tea occasionally, i.e., 2–4 days/week), and DTD (the consumers drinking tea almost daily, i.e., 5–7 days/week), according to their tea-drinking frequency. For diversified consumer demands, we optimized the brewing conditions of *Tieguanyin* based on the obtained response surface models, with a combined consideration of professional specialists and ordinary tea consumers.

## Results and discussion

### The sensory traits of *Tieguanyin* oolong tea infusion under different brewing conditions

**Fig. 1:**
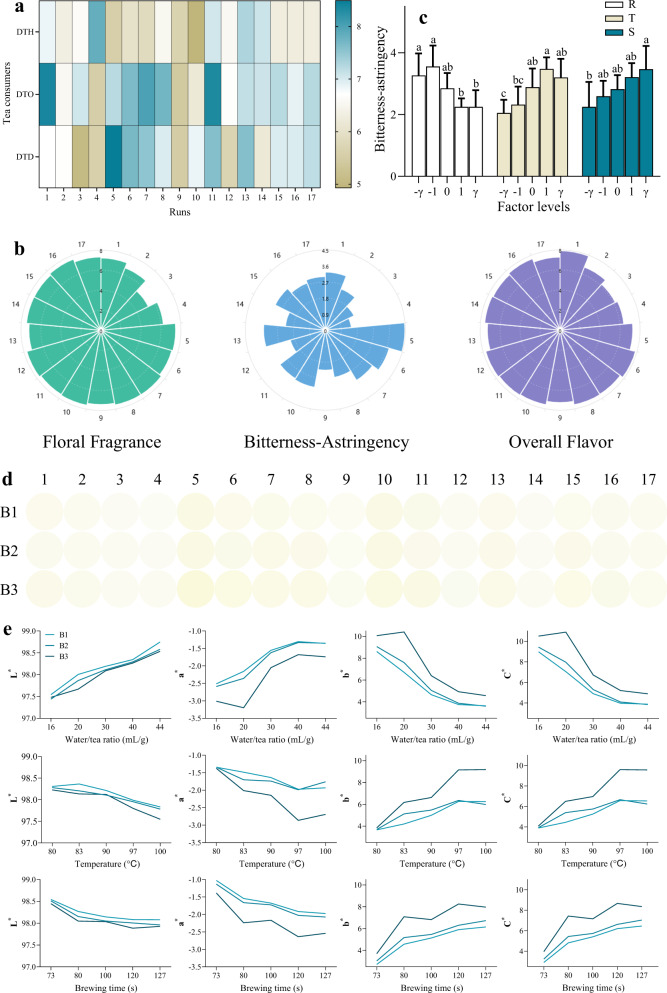
The sensory traits of *Tieguanyin* oolong tea infusion. **a** The preference of tea consumers with different drinking habits; **b** Nightingale rose diagram of the overall sensory characteristics; **c** The effects of brewing conditions on bitterness-astringency of oolong tea infusion; **d** The general situation of chromatic aberration for all the tea infusions; **e** The trends of each chromatic aberration index under different brewing conditions. DTH/DTO/DTD: the consumers drinking tea 0–1 (Hardly), 2–4 (Occasionally), 5–7 (Almost daily) days a week, respectively. B1/2/3 the first/second/third brewing infusion, respectively. R water/tea ratio, T temperature, S brewing time. ^a,b,c,d^ Different letters above the column indicate significant differences between different factor levels. Data are means (±SD) of three replicates.

Tea consumers with different drinking habits showed distinct preferences for *Tieguanyin* oolong tea infusion under different brewing conditions (Fig. 1a). Among them, DTH preferred the tea infusion prepared with higher R, lower T, and shorter S, which was just opposite from DTD, whereas DTO were with a versatile preference. One-way ANOVA indicated that DTH and DTD groups were both sensitive to brewing factors (*p* < 0.01), but DTO not (*p* > 0.05). The favorite combination of brewing conditions was R = 40 mL/g, T = 83 °C, S = 73 s, for DTH, whereas for DTD the ideal condition was R = 20 mL/g, T = 100 °C, S = 127 s. Additionally, for the favorite brewing infusion, the first brewing infusion (B1) was preferred by DTH (70.59% of selection rate), while the second/third brewing (B2/3) was by DTO (71.37%) and DTD (64.25%), respectively. These might be associated closely with the distinct taste preferences of different consumers. According to what we learned from the consumers taking part in this study, DTH usually do not like to drink tea or coffee and are extremely sensitive to the bitterness and the astringency of drinks. Hence, they can perceive easily the slight change of bitterness or astringency of tea infusion, which is directly affected by brewing conditions. On the contrary, DTD are used to drinking tea every day. The bitterness and astringency of tea infusion, detestable to DTH, are what they are addicted to. However, DTO drink tea occasionally for different starting points, such as health care, refreshing, operational need, and so on. They generally have no marked aversions to bitterness or astringency, and thus do not really care about the taste of tea infusion within the normal concentration range. Therefore, DTH and DTD both showed obvious sensitiveness to brewing conditions whereas DTO do not, from the perspective of preference.

The sensory analysis based on a trained panel showed that the quality attributes of oolong tea infusion varied considerably, particularly for bitterness-astringency (Fig. 1b, c), which was significantly reduced by a higher R (*p* < 0.01) or lower T (*p* < 0.01)/S (*p* < 0.05). In general, the impacts from R and T were much more significant compared with S, among which floral fragrance, bitterness-astringency, as well as overall flavor, all increased with the lowering of R (*p* < 0.01) and the rising of T (*p* < 0.05). Furthermore, the number of brewing times also impacted the bitterness-astringency, and B1 had the lowest scores in bitterness-astringency, which explained well the preference towards B1 by the DTH group. Correlation analysis showed that the DTD’s preference for oolong tea infusion was significantly associated with some sensory attributes (floral fragrance: r = 0.545, *p* < 0.05; bitterness-astringency: r = 0.928, *p* < 0.0001; overall flavor: r = 0.492, *p* < 0.05). It indicated that DTD have a clear preference for a fuller or pungent mouthfeel with stronger bitterness and astringency, which is consistent with our speculations about the taste preference of DTD.

All the oolong tea infusions were distinct in appearance color as well, shown as Fig. 1d. Among those color attributes (i.e., L^*^, a^*^, b^*^, C^*^), L^*^ was the least affected by brewing conditions, increased significantly only with R (r = 0.711, *p* < 0.001) (Fig. 1e). Among the three brewing factors, R exerted the greatest impact on chromatic aberration indexes, whereas the impact from S was insignificant. Specifically, R influenced positively on L^*^ and a^*^ (r = 0.662, *p* < 0.01) of tea infusion, while negatively on b^*^(r = −0.686, *p* < 0.01) and C^*^ (r = −0.685, *p* < 0.01). In addition, the number of brewing times impacted significantly the instrumental color (i.e., a^*^, b^*^, C^*^) whereby B1 presented the lightest color compared with the other two brewing infusions, which indicated that the first brewing tea infusion was with the lowest concentration.

### Effect of brewing conditions on the chemical composition of *Tieguanyin* oolong tea infusion

**Fig. 2:**
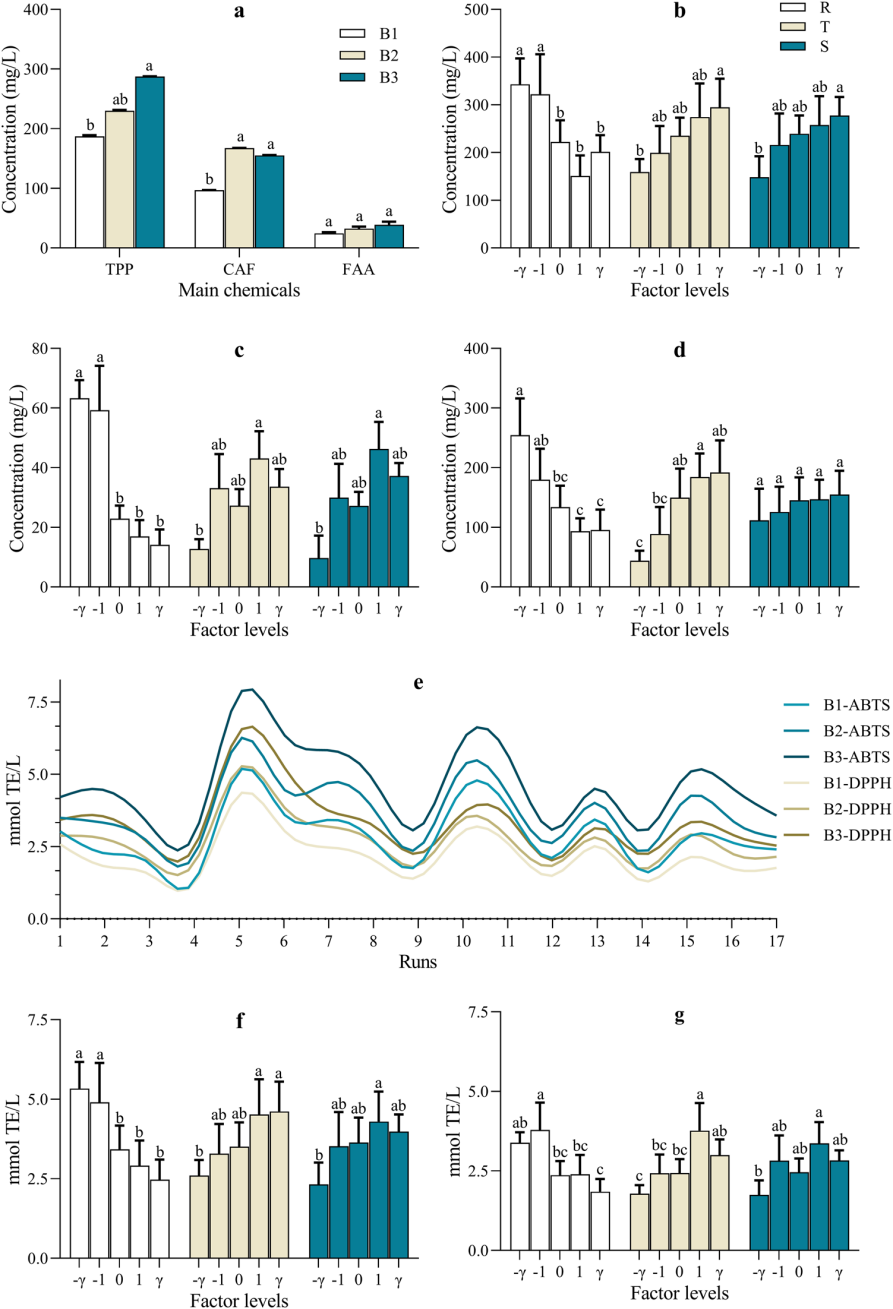
The main chemicals and antioxidant activity of *Tieguanyin* oolong tea infusion. **a** The effects of number of brewing times on the main chemicals; **b–d** The effects of brewing conditions on the concentrations of TPP, FAA, and CAF in tea infusions, respectively; **e** The antioxidant activity of tea infusions; **f, g** The effects of brewing conditions on the antioxidant activity in ABTS/DPPH assays, respectively. B1/2/3 the first/second/third brewing infusion, respectively. TPP total polyphenols, FAA free amino acids, CAF caffeine. R water/tea ratio, T temperature, S brewing time. ^a,b,c,d^ Different letters above the column indicate significant differences between different factor levels. Data are means (±SD) of three replicates.

The main chemicals of oolong tea infusion, including total polyphenols (TPP), caffeine (CAF), and free amino acids (FAA), were influenced greatly by the selected brewing factors, as shown in Fig. 2a–d. The results from Fig. 2a suggested that the extraction of TPP and CAF were significantly influenced by the number of brewing times. The yield and leaching rate of B1 were both obviously lower (*p* < 0.05) than those of the other two brewing infusions (i.e., B2/3), with the leaching rate of B2 being the highest whereas the yield of B3 being the highest. In this study, each brewing run began with the first brewing for which dry tea leaves were soaked in hot water, after separating and obtaining the first brewing tea infusion, the soaked tea leaves were used for the second and third brewing sequentially. During the whole process, the extraction was much more difficult for B1 than the other two, because dry tea leaves need to absorb enough water before releasing components. Then, the abundant components began to transfer from soaked tea leaves to infusion at a high speed for B2, which caused the highest leaching rates of TPP and FAA for B2. Finally, after B2, the leaching rate descended for B3, but the yield ranked first due to the longer T. However, the leaching of FAA was almost unaffected and extracted stably under the three brewing times. In a previous study of green tea, the concentration of FAA was also found to be impacted, by extraction temperature and time, much less compared with other components (TPP, CAF, etc.)^17^. The low molecular weight of FAA was considered the main cause, with that FAA could pass through the cell membrane of the tea leaves easily.

Likewise, R provided the greatest impact on the extracting yield compared to the other brewing factors, while S was just the complete opposite (Fig. 2b–d). Specifically, R acted on all the three types of chemicals with almost the same pattern in that the yield and leaching rate were both reduced significantly with R rising. As for T, it mainly worked positively on CAF (Fig. 2d). That’s to say, CAF is much more sensitive to water temperature, and a higher one is helpful for its extracting, which is in agreement with the previous researches^18,19^. Thus, brewing tea under a lower temperature or in cold water would be a good choice for caffeine intolerant consumers.

TPP is recognized as the most abundant and important component of teas. Its main composition (i.e., gallic acid (GA) and catechins) was further investigated by HPLC (Supplementary Table 2). Those components, except for GA, were influenced by brewing conditions nearly the same as TPP, where R decreased but T increased the extraction of tea catechins. Unlike catechins, GA was extracted regardless of the brewing conditions, except for S. A higher extracting yield of GA in tea infusion was obtained in longer S (*p* < 0.01). In addition, there was a significant correlation (r = 0.938, *p* < 0.01) between the total catechins and TPP, confirming that catechins are the main polyphenols in oolong tea infusion.

### Antioxidant activity of *Tieguanyin* oolong tea infusion under different brewing conditions

The antioxidant capacity, a simple but important health-related index for tea drinks, was measured by ABTS and DPPH assays, respectively, in this study. Shown as Fig. 2e, the antioxidant capacity of *Tieguanyin* oolong tea infusion varied greatly among all the runs. It was easy to find that the antioxidant activity enhanced gradually with increasing the number of brewing times, and B3, with the most TPP (Fig. 2a), had the highest antioxidant potential. R and T also had significant effects on the antioxidant activity of tea infusion as shown in Fig. 2f, g. A higher R, or lower T, led to the lower antioxidant activity of tea infusion, which was in line with the results for TPP and catechins. To be specific, the antioxidant activity of tea infusion brewed at the R levels of −*γ* (16 mL/g) and −1 (20 mL/g) were the highest, whereas the T level of −*γ* (83 °C) was the lowest. Correlation analysis revealed that the antioxidant activity of oolong tea infusion was associated positively with the concentrations of TPP (ABTS: r = 0.955, *p* < 0.01; DPPH: r = 0.897, *p* < 0.01) and total catechins (ABTS: r = 0.964, *p* < 0.01; DPPH: r = 0.967, *p* < 0.01). These implied that the *Tieguanyin* oolong tea infusion, brewed under the conditions of lower R or higher T, contained much more TPP and possessed higher antioxidant capacity, which would attract quite the interest of some consumers who pursue health. As many researches have reported, polyphenols, exactly catechins, are the predominant contributors to the antioxidant capacity of tea^3,12,20^.

### Principal component analysis (PCA)

**Fig. 3:**
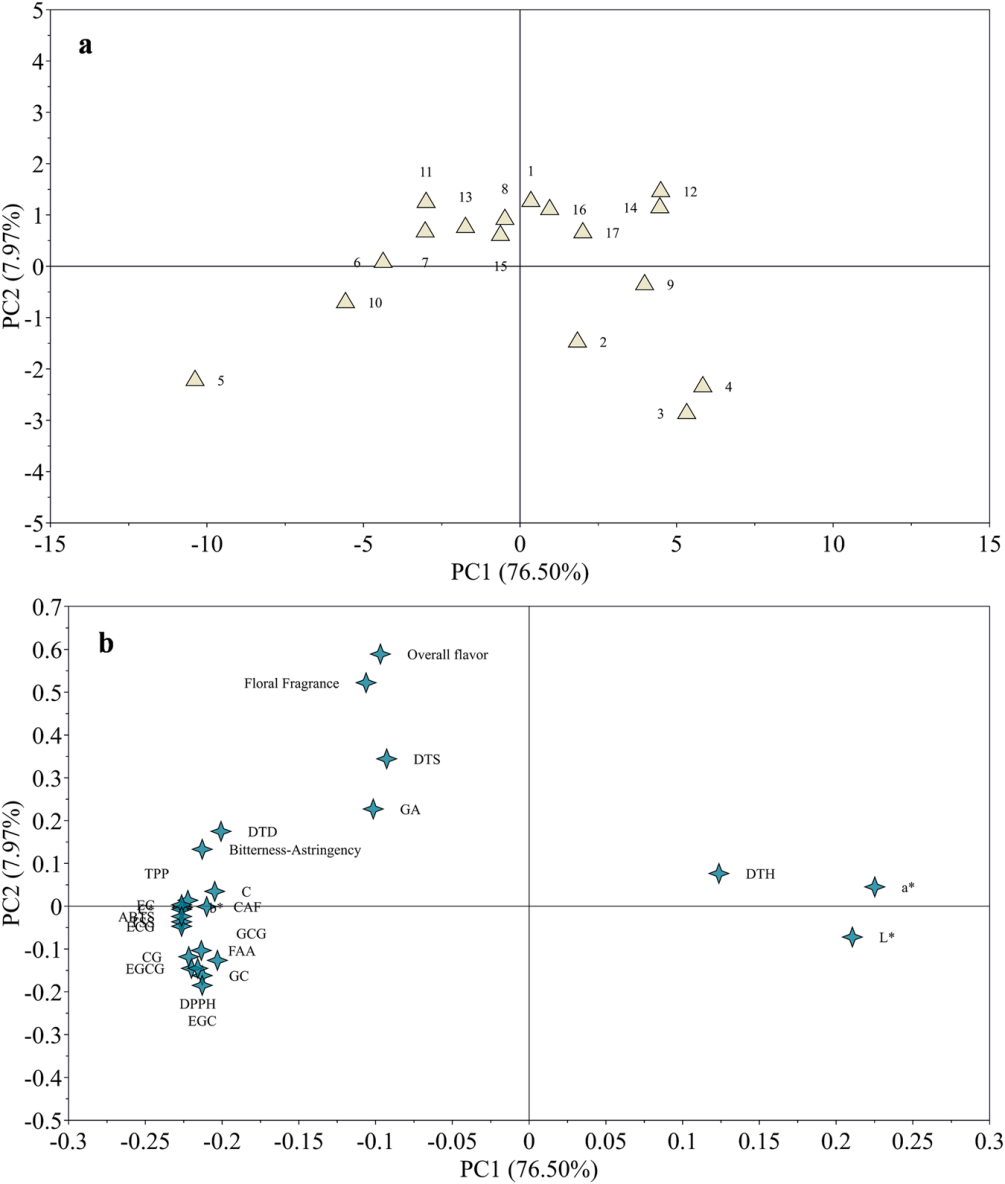
The PCA analysis of the sensory traits, chemical composition, and antioxidant activities of *Tieguanyin* oolong tea infusion. **a** The score plot; **b** The loading plot. DTH/DTO/DTD: the consumers drinking tea 0–1 (Hardly), 2–4 (Occasionally), 5–7 (Almost daily) days a week, respectively. TPP total polyphenols, FAA free amino acids, CAF caffeine. ABTS/DPPH the antioxidant activity of tea infusion in ABTS/DPPH assays, respectively.

Principal component analysis (PCA, Fig. 3) was applied to illustrate the complex relationships among the sensory quality traits, chemical composition, and antioxidant activity of oolong tea infusion. The score plot (Fig. 3a) presented the distribution of all the assays in the factor plane, while the loading plot (Fig. 3b) showed all the responses. It was found that the marks of all the chemicals contained in tea infusion and antioxidant activity (i.e., ABTS and DPPH) were gathered on the offside of the grid, far from those of “DTH”, “L^*^”, and “a^*^” locating on the other side. It indicated that the lightness and redness were negatively correlated to all these components, and DTH had a lower preference for the tea infusion with abundant chemicals and strong antioxidant activity. Obviously, more chemicals in tea infusion, mainly TPP and catechins, can cause more intensive bitterness-astringency, which may impair the preference for such tea infusion. Besides, rich components in tea infusion were beneficial for its floral fragrance and overall flavor, to a certain degree, but excessive content was inadvisable. In addition, the floral fragrance was associated closely with the overall flavor of tea infusion, while bitterness-astringency showed dual effects. In addition, the overall flavor evaluated by the trained taste panel was found to be not entirely consistent with the preference of tea consumers. Results from consumer tests and those obtained by a taste panel were different but should be considered concomitantly, because they provide unique insights on the quality attributes, such as intensity of flavor, bitterness/astringency, and degree of liking of color and taste. Therefore, similar tests (e.g., preference, acceptance test) focusing on consumers are extremely important for the flavor study in the tea-drinking field, which is exactly what previous studies lacked.

### Optimization of brewing conditions for *Tieguanyin* oolong tea based on response surface methodology (RSM)

**Fig. 4:**
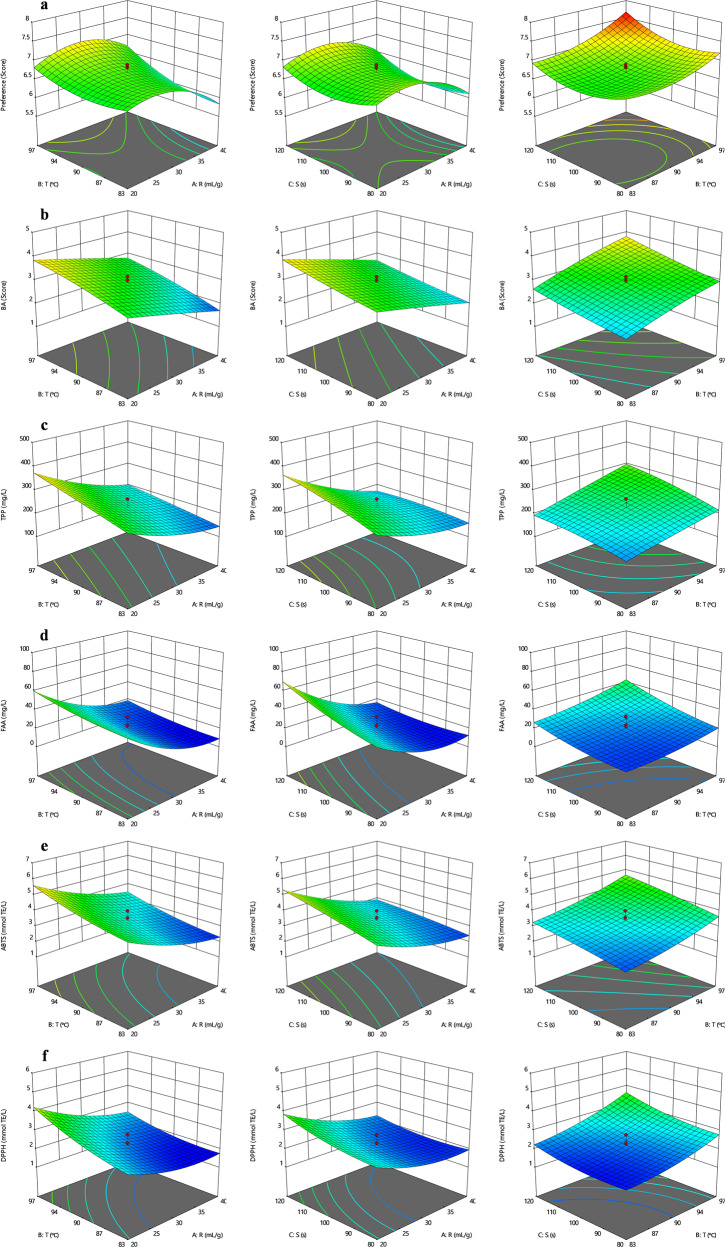
The response surface regression analysis. **a–f** The effects of brewing conditions on preference (**a**), bitterness-astringency (**b**), TPP (**c**), FAA (**d**), ABTS (**e**), and DPPH (**f**). TPP total polyphenols, FAA free amino acids. ABTS/DPPH the antioxidant activity of tea infusion in ABTS/DPPH assays, respectively.

Using response surface methodology (RSM), mathematical models were proposed based on quadratic orthogonal regression design to optimize the brewing conditions of *Tieguanyin* oolong tea. Those response surface models were put in Fig. 4. The specific parameters for the models were disclosed in Supplementary Table 3, suggesting that all the models fitted well (*p* < 0.05, R^2^ > 0.856) and were able to be used for optimization. The optimal brewing schemes depending on diversified consumer demands were listed in Table 1, including those special for tea consumers with different drinking habits (i.e., DTH, DTO, and DTD) and seeking flavor and health benefits at the same time. The brewing condition of R = 34 mL/g, T = 80 °C, S = 80 s, was obtained for DTH or the tea consumers seeking extremely light flavor, whereas the condition of R = 39 mL/g, T = 100 °C, S = 127 s was aimed at DTO and those like tea infusion with a mild taste. DTD group, with a preference for tea infusion with a strong flavor, would be satisfied with the tea infusion brewed under the condition of R = 20 mL/g, T = 100 °C, S = 100 s. Additionally, for the common consumers seeking flavor and health benefits meanwhile, R = 26 mL/g, T = 100 °C, and S = 127 s would be the most appropriate choice.

**Table 1:** The optimization of brewing conditions based on different consumer demands and predicted and experimental values for the optimized response variables.

Solution	Consumer demand	Optimal brewing condition			Response variable^a^	Predicted value^b^	Experimental value^c^	Significance^d^
		R (mL/g)	T (°C)	S (s)				
1	For DTH or consumers pursing light flavor	34	80	80	DTH	7.95 ± 0.41	8.52 ± 1.64	ns
2	For DTO or consumers pursing mild flavor	39	100	127	DTO	9.31 ± 0.48	9.07 ± 1.30	ns
3	For DTD or consumers pursing strong flavor	20	100	100	DTD	7.94 ± 0.43	7.93 ± 1.05	ns
4	For common consumers pursing flavor and health benefits	26	100	127	Preference	8.27 ± 0.30	7.54 ± 1.93	ns
					ABTS^e^	5.91 ± 0.38	6.99 ± 0.61	ns
					DPPH^e^	5.01 ± 0.41	4.44 ± 0.21	ns

^a^DTH/DTO/DTD: the consumers drinking tea 0–1 (Hardly), 2–4 (Occasionally), 5–7 (Almost daily) days a week, respectively.

^b^The predicted value is presented as “predicted mean ± standard deviation”.

^c^The experimental value is presented as “experimental mean ± standard deviation”.

^d^The marks show the significance of differences between predicted and experimental values, ns indicates *p* > 0.05, * indicates *p* < 0.05.

^e^The antioxidant activity values of ABTS and DPPH are expressed as mmol TE/L.

To verify the accuracy of the models, we carried out an external validation procedure using the optimized parameters to brew the same *Tieguanyin* oolong tea. The validation results (Table 1) showed that the experimental values for all the response variables were not different significantly (*p* < 0.05) from the predicted ones calculated from the models, which indicated a good prediction of those models.

## Conclusion

In this study, different brewing factors—R, T, and S—were selected and designed to optimize the brewing of *Tieguanyin* oolong tea using a quadratic orthogonal regression design. Results showed that these three brewing factors affected significantly the sensory traits, chemical composition, and antioxidant activity of *Tieguanyin* oolong tea infusion, especially R and T. A higher R, or lower T, would bring out a tea infusion with less bitterness-astringency, bioactive compounds and antioxidant activity, and a lighter color. Ordinary tea consumers with different drinking habits showed distinct preferences for a tea infusion, in a way that DTH preferred an infusion with a mild taste, while DTD showed the opposite behavior, and DTO presented a more versatile preference. Based on inconsistencies between the professional evaluation from specialists with the preference presented by consumers, we optimized the brewing conditions for different consumer segments in RSM and validated these findings.

## Methods

### Chemical compounds

(+)-Catechin (C), (−)-gallocatechin (GC), (−)-catechin gallate (CG), (−)-gallocatechin gallate (GCG), (−)-epicatechin (EC), (−)-epigallocatechin (EGC), (−)-epicatechin gallate (ECG), (−)-epigallocatechin gallate (EGCG), caffeine, gallic acid (GA), glutamic acid, Folin-Ciocalteu’s phenol, acetonitrile, potassium persulfate (K_2_S_2_O_8_), 2, 2-diphenyl-1-picrylhydrazyl (DPPH), 2, 2’-azino-bis (3-ethylbenzothiazoline-6-sulfonic acid) (ABTS), and 6-hydroxy-2, 5, 7, 8-tetramethylcoromane-2-carboxylic acid (Trolox) were from Sigma–Aldrich (Shanghai, China). Acetic acid, methanol, and stannous chloride (SnCl_2_·2H_2_O) were from Aladdin Co., Ltd (Shanghai, China). Sodium carbonate (Na_2_CO_3_), potassium dihydrogen phosphate (KH_2_PO_4_), and dibasic sodium phosphate (Na_2_HPO_4_) were from Macklin Biochemical Co., Ltd. (Shanghai, China). 2, 2-Dihydroxyindane-1, 3-dione (ninhydrin) was from Alfa Aesar Co., Ltd. (Shanghai, China).

### Tea sample and preparation of tea infusion

*Tieguanyin* oolong tea sample, belonging to the light-scented type, was obtained from the Tea Research Institute of Chinese Academy of Agricultural Sciences (TRICAAS; Hangzhou, China), which was the same as the sample we used in our previously study^3^. The brewing water used was pure water from Hangzhou Wahaha Group Co., Ltd. (Hangzhou, China).

Quadratic orthogonal regression design (p = 3, m_0_ = 3, *γ* = 1.353) was employed, using Design Expert (Version 11, State-Ease, Minneapolis, MN, USA), to study the effect of brewing conditions on *Tieguanyin* oolong tea infusion. Experiment assays were shown in Table 2 and contained three factors (R, T, and S) with five levels (Supplementary Table 1). To simulate the situation of daily tea drinking, a brewing method consisting of three successive extractions was adopted^3^. The extraction time for each brewing was specified in Supplementary Table 1, among which the second/third brewing time (i.e., S2/S3) totally depended on the first one (i.e., S1). Each tea infusion (150 mL) was prepared with pure water under different brewing conditions according to Table 2, at room temperature (RT, 25 ± 2 °C), and repeated three times on different days.

**Table 2:** Brewing experiment arrangements based on quadratic orthogonal regression design (*p* = 3, m_0_ = 3, *γ* = 1.353).

Points	Runs	Factors (code values (actual values))		
		Water/tea ratio (mL/g)	Temperature (°C)	Time (s)
		R	T	S
Orthogonal points (m_c_ = 2^3^ = 8)	1	1 (40)	1 (97)	1 (120)
	2	1 (40)	1 (97)	−1 (80)
	3	1 (40)	−1 (83)	1 (120)
	4	1 (40)	−1 (83)	−1 (80)
	5	−1 (20)	1 (97)	1 (120)
	6	−1 (20)	1 (97)	−1 (80)
	7	−1 (20)	−1 (83)	1 (120)
	8	−1 (20)	−1 (83)	−1 (80)
Axial points (2p = 6)	9	*γ* (44)	0 (90)	0 (100)
	10	−*γ* (16)	0 (90)	0 (100)
	11	0 (30)	*γ* (100)	0 (100)
	12	0 (30)	−*γ* (80)	0 (100)
	13	0 (30)	0 (90)	*γ* (127)
	14	0 (30)	0 (90)	−*γ* (73)
Central points (m_0_ = 3)	15	0 (30)	0 (90)	0 (100)
	16	0 (30)	0 (90)	0 (100)
	17	0 (30)	0 (90)	0 (100)

### Sensory preference test and evaluation of tea infusion

A sensory preference test was conducted among ordinary tea consumers (11 men, 16 women, 22–45 years old) with different drinking habits. Those drinking tea 0–1, 2–4, 5–7 days a week were labeled as DTH (the consumers drinking tea hardly), DTO (the consumers drinking tea occasionally), and DTD (the consumers drinking tea almost daily), respectively. The preference for each tea was scored on a 10-point scale modified from Xu et al.^4^, and the greater value (0–10) implied a higher sensory preference. Besides, the favorite one of each tea was required to be selected among the three brewing tea infusions (i.e., B1, B2, B3).

The sensory attributes, i.e., floral fragrance, bitterness-astringency, and overall flavor, of tea infusion were evaluated by five trained panelists (3 men, 2 women, 25–48 years old) on 10-point scale, according to the method used in our previous studies^3,4^.

All the participants (healthy and nonsmokers from TRICAAS) were conducted in accordance with the principle set forth in the Declaration of Helsinki and informed written consent was obtained. This study was approved by the Zhejiang Gongshang University Human Ethics Committee. All the participants have been asked not to eat or drink anything except for pure water one hour before the experiment. All the experiments were conducted at RT, and tea infusions were treated with a water bath (45 °C), which is close to daily drinking, during the whole process.

### Analysis of chromatic parameters (color) of tea infusion

The instrumental color of each tea infusion was analyzed in a spectrophotometer (CM-3500d, Konica Minolta (China) Investment Ltd., Shanghai, China), as previously described^21^.

### Determination of chemical composition in tea infusion

The total polyphenols (TPP) and free amino acids (FAA) in tea infusion were determined with Folin-Ciocalteu^22^ and ninhydrin colorimetric^23^ assays, respectively. A spectrophotometer (Spectrophotometer 2000, Unico (Shanghai) Instrument Co., Ltd, Shanghai, China) was used after zero calibration with pure water.

The analysis of gallic acid (GA), caffeine (CAF), and catechins was performed by high-performance liquid chromatography/UV detection (HPLC/UV; Shimadzu, Tokyo, Japan) using the conditions described elsewhere^3^. The specific method was as follows: Diamonsil™ C18 column (4.6 × 250 mm, 5 μm; Dikma Technologies Inc., Lake Forest, CA); the mobile phases A/B: 2% acetic acid/100% acetonitrile; injection volume 10 μL; flow rate 1.0 mL/min; column temperature 35 °C; detection wavelength 280 nm; post-run time 5 min. The elution solvent was initially 6.5% B, then ramped linearly to 15% B at 16 min, held at 15% B until 25 min, then ramped back to 6.5% B at 30 min.

### Determination of the antioxidant activity of tea infusion

The antioxidant activity of tea infusion was measured in triplicate using ABTS and DPPH assays, as detailed fully in a previous study^24^, with Trolox as the reference standard, and the results were expressed as mmol Trolox equivalents/L (mmol TE/L).

#### DPPH assay

The samples of tea infusion, Trolox standard solution, or methanol (blank) of 100 μL were added into DPPH stock solution (25 mg/L in methanol) of 3.9 mL, and the mixtures were incubated in dark for 2 h at RT. After that, the mixed solutions were measured at 515 nm using the spectrophotometer.

#### ABTS assay

The ABTS reaction solution was prepared by the reaction of ABTS solution (7 mM) with K_2_S_2_O_8_ (2.45 mM) in equal volume, and kept in dark for 12–16 h. Then the ABTS reaction solution needs to be diluted in methanol to an absorbance of 0.70 (± 0.02) before use. The reaction system consisted of 50 μL of tea infusion, Trolox standard solution, or methanol (blank) with 2 mL of ABTS reaction solution. The mixtures were equilibrated for 7 min before absorbance measurement at 734 nm in the spectrophotometer.

### Statistical analysis

All the data were presented as mean ± standard deviation (SD), in triplicate. The analysis of the significant difference between the means was carried out by one-way analysis of variance (ANOVA), followed by Duncan’s multiple range test, to compare the means for significant variation (*p* < 0.05). Correlation analysis between attributes was conducted using Pearson’s correlation coefficients. Principal component analysis (PCA) was conducted in Simca-P (Version 14.1, MKS Umetrics AB, Umeå, Sweden), response surface methodology (RSM) in Design Expert (Version 11, State-Ease, Minneapolis, MN, USA), the other statistical analyses in SAS software (Version 9.4, SAS Institute Inc., Cary, NC, USA). Figures were plotted in GraphPad Prism (Version 9.00, GraphPad Software Inc., San Diego, CA, USA).

## Supplementary information

Supplementary Material-0330

## Data Availability

The datasets produced from the study are available from the corresponding author on reasonable request.
