# Supplementary material for: Optimization of brewing conditions for Tieguanyin oolong tea by quadratic orthogonal regression design
Source: NPJ Sci Food. 2022 Apr 25;6:25. doi: 10.1038/s41538-022-00141-7 (PMC9038793; doi:10.1038/s41538-022-00141-7)
Supplement: Supplementary file 1 — Supplementary Material-0330 [file 41538_2022_141_MOESM1_ESM.pdf]

Optimization of brewing conditions for *Tieguanyin* oolong tea by quadratic orthogonal regression design

Qing-Qing Cao [a, b], Jie-Qiong Wang [a, b], Jian-Xin Chen [a], Fang Wang [a], Ying Gao [a *], Daniel Granato [c], Xuebo Zhang [d], Jun-Feng Yin [a], Yong-Quan Xu [a **]

[a]*Tea Research Institute Chinese Academy of Agricultural Sciences, Key Laboratory of Tea Biology and Resources Utilization, Ministry of Agriculture, 9 South Meiling Road, Hangzhou 310008, China*

[b]*Graduate School of Chinese Academy of Agricultural Sciences, Beijing 100081, China*

[c]*Department of Biological Sciences, Faculty of Science and Engineering, University of Limerick, V94 T9PX Limerick, Ireland. E-mail:* daniel.granato@ul.ie

[d]*National Tea Quality Supervision and Inspection Center (Fujian), Anxi 362400, China.*

Corresponding Authors

[*]Yong-Quan Xu, Tel: +86-571-86650594. Fax: +86 571 86650056. Email: yqx33@126.com.

Table 1. Brewing conditions setting based on quadratic orthogonal regression design

Factor / Level	Water/tea ratio (mL/g)	Temperature (°C)	Time (s)		
	R	T	S1	S2	S3
-γ	16	80	73	63	103
-1	20	83	80	70	110
0	30	90	100	90	130
1	40	97	120	110	150
γ	44	100	127	117	157

S1/2/3: the brewing time for the first/second/third brewing infusion, respectively.

Table 2. The concentrations of GA and catechins in *Tieguanyin* oolong tea infusions

Runs	Concentration (mg/L)-B1								
	GA	GC	EGC	C	EGCG	EC	GCG	ECG	CG
1	1.62±0.01	13.75±0.32	62.73±0.61	1.34±0.03	55.16±2.06	16.62±0.08	0.94±0.06	11.89±0.67	0.48±0.02
2	1.13±0.00	10.80±0.21	53.44±0.15	1.36±0.02	46.14±2.22	14.18±0.13	0.78±0.05	9.84±0.72	0.42±0.02
3	3.08±3.91	7.60±0.50	41.13±6.57	0.81±0.12	27.49±8.75	8.87±0.60	0.40±0.10	5.40±1.99	0.31±0.02
4	0.66±0.00	7.27±0.08	34.41±0.25	0.45±0.01	27.11±2.22	7.44±0.08	0.36±0.03	5.37±0.62	0.27±0.00
5	1.75±0.03	30.44±0.61	135.28±0.62	2.19±0.02	121.51±2.61	31.94±0.04	1.86±0.05	24.71±0.75	0.96±0.03
6	1.36±0.03	16.52±0.56	83.94±0.49	1.30±0.06	73.38±1.84	20.40±0.03	1.25±0.06	15.10±0.73	0.66±0.01
7	2.85±0.03	10.81±0.33	69.19±4.04	1.34±0.03	64.72±0.88	19.93±0.55	0.74±0.04	14.67±0.47	0.56±0.03
8	2.13±0.04	9.37±0.31	52.29±1.62	1.19±0.03	45.21±2.22	14.68±0.24	0.50±0.06	10.42±0.77	0.48±0.01
9	1.32±0.09	6.45±0.94	27.10±8.29	1.37±0.05	24.04±5.12	10.56±0.42	0.38±0.07	6.88±0.64	0.47±0.24
10	3.05±0.69	19.20±0.07	99.02±1.82	1.94±0.03	88.62±1.87	27.98±0.25	1.12±0.05	19.80±0.79	0.77±0.02
11	2.64±0.10	13.68±0.17	70.25±2.41	2.44±0.11	68.57±0.88	26.68±0.25	1.08±0.04	17.34±0.81	0.57±0.01
12	1.70±0.18	10.39±2.30	12.93±5.02	0.78±0.07	16.33±3.77	10.32±0.18	0.38±0.04	7.39±0.54	0.28±0.00
13	2.00±0.54	13.21±0.08	80.04±0.53	1.68±0.02	73.26±2.51	21.57±0.14	0.87±0.04	16.17±0.74	0.58±0.00
14	1.05±0.04	5.39±0.23	31.17±0.76	0.67±0.02	28.47±2.51	8.79±0.12	0.37±0.04	6.09±0.66	0.27±0.00
15	2.63±0.51	8.99±0.36	49.50±0.87	1.34±0.03	44.71±0.17	15.55±0.11	0.57±0.01	10.79±0.15	0.43±0.01
16	2.00±0.03	12.24±1.17	22.48±1.36	1.48±0.23	24.69±2.70	13.42±0.38	0.61±0.05	8.89±0.74	0.41±0.01
17	1.47±0.07	7.88±0.31	47.98±0.80	1.13±0.05	41.00±2.18	13.47±0.18	0.53±0.04	9.37±0.70	0.40±0.00

Runs	Concentration (mg/L)-B2								
	GA	GC	EGC	C	EGCG	EC	GCG	ECG	CG
1	1.55±0.01	13.55±0.88	69.36±1.16	1.75±0.01	63.79±1.57	20.60±0.32	1.25±0.05	14.67±0.62	0.49±0.05
2	1.21±0.01	13.39±0.56	71.55±0.63	1.75±0.13	61.70±2.00	19.74±0.05	1.10±0.06	13.37±0.74	0.49±0.02
3	0.84±0.01	9.81±0.29	51.40±0.67	1.16±0.02	44.20±2.25	11.67±0.07	0.58±0.03	8.83±0.60	0.38±0.01
4	0.85±0.01	10.41±0.85	56.07±1.47	1.00±0.04	45.51±1.69	11.98±0.11	0.60±0.04	9.11±0.52	0.38±0.03
5	1.70±0.04	34.45±0.51	164.04±0.50	3.09±0.16	155.72±2.50	42.12±0.04	2.60±0.06	33.00±0.79	1.11±0.06
6	3.44±3.30	20.82±0.65	110.84±4.81	2.01±0.24	92.58±5.27	30.37±0.76	1.64±0.06	19.99±1.39	0.75±0.02
7	3.36±0.04	17.27±0.69	112.47±0.17	1.92±0.09	105.27±3.52	30.83±0.42	1.12±0.08	23.25±0.96	0.79±0.02
8	2.82±0.11	14.85±0.14	81.31±1.32	1.80±0.06	71.98±1.79	22.36±0.23	0.63±0.01	16.57±0.79	0.70±0.01
9	1.29±0.07	8.13±0.24	45.21±1.47	1.46±0.03	39.21±3.36	15.00±0.27	0.50±0.05	9.65±0.85	0.40±0.01
10	3.49±0.16	22.60±0.23	120.32±0.58	2.59±0.02	107.10±2.92	36.19±0.08	1.38±0.05	24.57±0.85	0.89±0.00
11	2.81±0.11	17.57±0.46	62.00±2.34	3.16±0.02	67.29±2.63	31.92±0.27	1.48±0.05	20.40±0.81	0.54±0.02
12	1.89±0.05	11.90±0.46	19.72±2.70	1.11±0.02	24.36±3.06	13.64±0.23	0.50±0.05	9.65±0.74	0.33±0.00
13	2.22±0.06	14.36±0.80	92.81±0.22	2.20±0.03	89.48±2.93	26.94±0.24	1.24±0.06	20.44±0.90	0.59±0.00
14	1.11±0.13	7.36±0.48	43.92±2.59	1.12±0.25	40.69±3.53	13.03±0.25	0.54±0.04	9.03±0.70	0.31±0.00
15	2.91±0.46	14.07±0.75	82.11±1.72	2.10±0.07	80.65±2.22	25.17±0.71	0.92±0.04	19.21±0.83	0.62±0.01
16	1.96±0.10	13.60±1.16	42.71±5.78	1.67±0.05	41.77±4.70	18.46±0.63	0.84±0.03	12.46±0.34	0.49±0.01
17	1.64±0.17	9.87±0.19	62.23±1.60	1.53±0.04	53.86±2.66	19.18±0.54	0.68±0.04	12.63±0.77	0.45±0.00

Runs	Concentration (mg/L)-B3								
	GA	GC	EGC	C	EGCG	EC	GCG	ECG	CG
1	1.51±0.01	17.22±0.47	85.64±0.47	2.27±0.02	83.98±2.49	24.39±0.12	1.98±0.07	19.18±0.76	0.65±0.01
2	1.41±0.28	16.44±0.10	90.44±0.23	2.24±0.03	82.35±2.11	23.97±0.19	1.83±0.07	17.91±0.66	0.61±0.02
3	0.90±0.01	12.48±0.39	66.05±0.73	1.48±0.03	58.42±2.55	14.89±0.10	0.79±0.05	11.58±0.71	0.48±0.01
4	0.79±0.00	14.01±0.45	71.38±0.10	1.49±0.31	59.34±1.87	15.49±0.21	0.86±0.05	11.85±0.58	0.47±0.01
5	1.90±0.06	42.55±0.77	205.66±0.45	4.31±0.05	214.34±2.96	52.86±0.16	4.84±0.11	45.96±0.84	1.50±0.03
6	1.83±0.04	29.79±0.74	159.38±1.06	3.29±0.09	150.55±1.85	43.52±0.06	3.29±0.09	32.92±0.95	1.10±0.06
7	5.37±4.32	20.90±0.20	139.02±4.09	2.42±0.04	133.48±6.65	36.79±1.26	1.44±0.10	28.56±2.19	0.93±0.12
8	3.09±0.09	19.96±0.22	108.02±1.47	2.52±0.03	97.86±2.20	29.84±0.30	0.88±0.02	22.40±0.86	0.91±0.02
9	1.42±0.02	10.16±0.20	55.54±1.38	1.90±0.06	51.77±2.14	18.99±0.12	0.83±0.06	12.97±0.83	0.50±0.01
10	3.86±0.46	26.33±0.19	140.24±0.37	3.27±0.01	130.24±3.51	42.82±0.13	2.09±0.05	30.24±1.09	1.04±0.00
11	2.97±0.19	22.26±0.22	102.82±3.04	4.23±0.32	115.18±3.67	39.46±0.34	3.38±0.12	29.85±0.92	0.77±0.01
12	1.99±0.11	12.12±1.09	27.08±2.23	1.31±0.02	32.03±2.15	15.92±0.28	0.62±0.04	11.60±0.78	0.36±0.01
13	14.91±22.04	18.19±2.08	123.34±31.60	2.66±0.01	85.60±33.34	32.75±4.26	1.55±0.42	18.93±8.27	0.71±0.04
14	1.47±0.08	10.42±0.15	65.58±0.27	1.64±0.01	62.22±2.68	19.23±0.22	0.86±0.05	13.12±0.76	0.40±0.00
15	2.80±0.22	17.51±0.70	94.54±8.12	2.64±0.04	99.41±7.11	30.31±0.87	1.54±0.10	23.78±0.84	0.78±0.07
16	2.35±0.02	18.23±0.46	72.72±4.81	2.48±0.02	68.69±3.84	26.64±0.26	1.38±0.06	17.90±0.81	0.72±0.01
17	1.80±0.09	12.18±0.24	78.70±1.13	2.11±0.12	70.10±3.33	24.72±0.72	1.09±0.15	16.39±0.88	0.53±0.01

Data are means (±SD) of three replicates.

Table 3. ANOVA of response surface regression model for the sensory profiles, phytochemical composition and antioxidant activities of *Tieguanyin* oolong tea infusion

Response variable	Factors	Regression coefficient		*p*-value
		Coded factors	Actual factors	
DTH	R^2	-0.533516173	-0.005335	0.0093
	T^2	0.225859855	0.004609	0.1658
	S^2	0.467052121	0.001168	0.0207
	RT	-0.06125	-0.000875	0.6818
	RS	0.05375	0.000269	0.7186
	TS	0.24875	0.001777	0.126
	R	0.396308725	0.411616	0.0118
	T	-0.112212838	-0.997148	0.3678
	S	-0.129368828	-0.407968	0.3119
	Intercept	6.28952559	65.83568	
	R^2	0.856166715	Model	0.0279
	adjusted R^2	0.671238205	Lack of fit	0.0734
DTO	R^2	-0.44821563	-0.004482156	0.0408
	T^2	0.39999497	0.008163163	0.0553
	S^2	0.252891287	0.000632228	0.2193
	RT	0.39875	0.005696429	0.0524
	RS	0.43875	0.00219375	0.0372
	TS	-0.04875	-0.000348214	0.7838
	R	-0.346812081	-0.497805402	0.0424
	T	0.316773649	-1.560187329	0.0569
	S	0.283211679	-0.146758274	0.0858
	Intercept	6.925601116	89.71481255	
	R^2	0.855640026	Model	0.0282
	adjusted R^2	0.670034346	Lack of fit	0.1169
DTD	R^2	-0.25753426	-0.002575343	0.146
	T^2	-0.028531717	-0.00058228	0.8576
	S^2	0.124198864	0.000310497	0.4759
	RT	0.13625	0.001946429	0.3949
	RS	-0.23375	-0.00116875	0.164
	TS	0.10625	0.000758929	0.5026
	R	-0.720469799	0.024170005	0.0006
	T	0.59535473	0.05557535	0.0018
	S	0.237612709	-0.083459868	0.0982
	Intercept	6.883424843	4.852369927	
	R^2	0.907397165	Model	0.0069
	adjusted R^2	0.788336378	Lack of fit	0.0518

Preference	R^2	-0.414007728	-0.004140077	0.007924618
	T^2	0.199847356	0.004078517	0.111161246
	S^2	0.282244908	0.000705612	0.048015684
	RT	0.15625	0.002232143	0.189718894
	RS	0.08375	0.00041875	0.461807367
	TS	0.10125	0.000723214	0.377988117
	R	-0.223825503	-0.016745771	0.038694033
	T	0.267584459	-0.835192509	0.01841493
	S	0.131644483	-0.212192016	0.183382312
	Intercept	6.700220459	53.43111193	
	R^2	0.865714741	Model	0.02253842
	adjusted R^2	0.693062266	Lack of fit	0.069708516
BA	R^2	-0.018887752	-0.000188878	0.860313141
	T^2	-0.086508057	-0.001765471	0.418609953
	S^2	0.039289411	9.82E-05	0.727515106
	RT	0.03375	0.000482143	0.742602047
	RS	-0.03125	-0.00015625	0.760930364
	TS	0.07125	0.000508929	0.49406166
	R	-0.558557047	-0.072290911	0.000230708
	T	0.519206081	0.326599854	0.000346963
	S	0.353542293	-0.043083662	0.003486165
	Intercept	2.911339728	-11.93088046	
	R^2	0.940010014	Model	0.001667341
	adjusted R^2	0.862880032	Lack of fit	0.218356085
TPP	R^2	21.54366612	0.215436661	0.127064274
	T^2	-1.365914383	-0.027875804	0.913364422
	S^2	-9.394840338	-0.023487101	0.49428502
	RT	-12.44875	-0.177839286	0.329595186
	RS	-23.56675	-0.11783375	0.087738662
	TS	12.1255	0.086610714	0.34145755
	R	-74.09885906	7.452825135	0.000124991
	T	40.85805405	7.528616679	0.003908363
	S	29.21889223	1.898412995	0.020899432
	Intercept	227.3204642	-542.738174	
	R^2	0.930899981	Model	0.002664805
	adjusted R^2	0.8420571	Lack of fit	0.610782136
FAA	R^2	10.66834345	0.106683435	0.008348958
	T^2	2.582055388	0.052695008	0.396042926
	S^2	3.189654408	0.007974136	0.333849297
	RT	-0.94275	-0.013467857	0.74642191
	RS	-7.0515	-0.0352575	0.040029627
	TS	2.62375	0.018741071	0.380309356

	R	-19.97994966	-3.661143895	5.30E-05
	T	5.761047297	-10.1321661	0.039443652
	S	8.812868184	-1.783155223	0.006766248
	Intercept	20.41972799	591.3445719	
	R^2	0.944153829	Model	0.001313175
	adjusted R^2	0.872351609	Lack of fit	0.827870281
CAF	R^2	14.02104008	0.140210401	0.008712991
	T^2	-14.36939684	-0.293252997	0.006767037
	S^2	-7.793314349	-0.019483286	0.09740962
	RT	-11.837875	-0.1691125	0.015367391
	RS	-5.831875	-0.029159375	0.160535196
	TS	0.625125	0.004465179	0.871159786
	R	-47.55657718	4.967780733	1.07E-06
	T	49.11858615	64.42933744	8.16E-07
	S	12.34098755	4.98662173	0.005144369
	Intercept	145.6624189	-3352.785506	
	R^2	0.987853273	Model	7.12E-06
	adjusted R^2	0.972236053	Lack of fit	0.325147187
ABTS	R^2	0.330624845	0.003306248	0.04944427
	T^2	0.172563434	0.003521703	0.243640932
	S^2	-0.050630492	-0.000126576	0.739151529
	RT	-0.07	-0.001	0.615018443
	RS	-0.21575	-0.00107875	0.148782067
	TS	0.05425	0.0003875	0.695661945
	R	-1.003808725	-0.100880779	3.68E-05
	T	0.647027027	-0.55022406	0.000555411
	S	0.459630743	0.045784283	0.004203619
	Intercept	3.388687995	23.56994499	
	R^2	0.954845278	Model	0.000643266
	adjusted R^2	0.896789206	Lack of fit	0.878735359
DPPH	R^2	0.341464319	0.003414643	0.061858056
	T^2	0.211775302	0.004321945	0.199879169
	S^2	0.197486209	0.000493716	0.259331591
	RT	-0.233375	-0.003333929	0.155806056
	RS	-0.165875	-0.000829375	0.29529531
	TS	0.109375	0.00078125	0.479767789
	R	-0.648473154	0.113265165	0.001014741
	T	0.585670608	-0.672390002	0.001743845
	S	0.312215543	-0.128563578	0.037193526
	Intercept	2.213789824	33.62768686	
	R^2	0.911306543	Model	0.006034745

	adjusted R^2	0.797272099	Lack of fit	0.366320259

DTH/DTO/DTD: the preference for consumers drinking tea 0-1 (Hardly), 2-4 (Occasionally), 5-7 (Almost daily) days a week, respectively; Preference: the preference for all the tea consumers consisting of DTH/DTO/DTD; BA: bitterness-astringency; TPP: total polyphenols; FAA: free amino acids; ABTS/DPPH: the antioxidant activity of tea infusion in ABTS/DPPH assays, respectively.
